# Multiple cranial neuropathy due to varicella zoster virus reactivation without vesicular rash: a challenging diagnosis

**DOI:** 10.1007/s10072-023-06833-6

**Published:** 2023-05-09

**Authors:** Antonio Stornaiuolo, Rosa Iodice, Roberto De Simone, Cinzia Russo, Marica Rubino, Simone Braca, Angelo Miele, Stefano Tozza, Maria Nolano, Fiore Manganelli

**Affiliations:** Department of Neurosciences, Reproductive Sciences and Odontostomatology, University Federico II of Naples, 80131 Naples, Italy

**Keywords:** Ramsay Hunt syndrome, VZV, Cranial neuropathy, Sine herpete, Zoster oticus

## Abstract

Ramsay Hunt syndrome is due to reactivation of varicella zoster virus (VZV) dormant in the geniculate ganglion of the facial nerve. The diagnosis is typically based on clinical triad of ipsilateral facial paralysis, otalgia, and vesicles in the auditory canal or the auricle. However, Ramsay Hunt syndrome may occur without skin eruption in up to one third of patients. Moreover, the involvement of other cranial nerves in addition to the facial nerve has been also reported. Herein, we reported a case report of a man who developed a multiple cranial neuropathy caused by VZV reactivation without skin vesicular eruption. The present case underlines a possible diagnostic challenge that clinicians may hit when facing a common disorder such as peripheral facial palsy. Indeed, clinicians must be aware that Ramsay Hunt syndrome may develop without skin vesicular eruption as well it may be complicated by multiple cranial nerve involvement. Antiviral therapy is effective in VZV reactivation for recovery of nerve function.

## Introduction

Ramsay Hunt syndrome, also known as herpes zoster oticus, was described by neurologist James Ramsay Hunt (1874–1937) and it is due to a reactivation of varicella zoster virus (VZV) in the geniculate ganglion of the facial nerve. Indeed, primary infection with VZV causes varicella while the reactivation of latent infection causes herpes zoster (shingles) that is a painful, blistering skin eruption in a dermatomal distribution. Ramsay Hunt syndrome accounts for less than 1% of all herpes zoster cases [1].

The diagnosis is typically based on clinical triad of ipsilateral facial paralysis, otalgia, and vesicles in the auditory canal or the auricle. Usually, the first symptom to appear is pain, followed after a variable period of 2–3 days by facial nerve palsy and vesicles. Additional symptoms may include altered taste sensation, dry eye, tearing, and hyperacusis as well vesicles may involve more widely the affected side of the face, scalp, palate, and tongue. However, herpes zoster without vesicles (called sine herpete) has been reported up to 30% of Ramsay Hunt cases [2].

Moreover, the involvement of other cranial nerves in addition to the facial nerve has been also reported. Tinnitus, hearing loss, and vertigo may be present if the vestibulocochlear nerve is involved [3], while hoarseness or aspiration may appear if the vagus nerve is compromised [4].

Herein, we reported a case report of a man who developed a multiple cranial neuropathy caused by VZV reactivation without skin vesicular eruption.

## Case report

A 77-year-old man went to the emergency department (ED) with left facial palsy, speech difficulty, and gait abnormalities. Three days earlier, he had consulted his physician for acute pharyngeal pain, accompanied by headache and left otalgia, and hence, he had started antibiotic therapy with Azythromycin (500 mg daily) without improvement. In the days to come, the pain became somewhat continuous and dull, of mild to moderate intensity, and responsive to Paracetamol 1000 mg. The patient located the pain in the temporal and preauricular region, not clearly superficial (as would be expected in case of neuralgic pain), while involvement of the ear canal was not reported. The pharyngodynia was not continuous, but the patient reported discomfort when swallowing solid food.

After the admission to ED, he underwent brain computed tomography (CT) and CT angiography that were negative for ischemic or haemorrhagic lesions and hence he was transported to neurology ward.

Neurological evaluation showed left upper and lower facial muscles paralysis that was consistent with a peripheral facial nerve palsy. However, clinical examination also revealed some gait instability, first grade right-sided nystagmus, mild dysarthria, mild paralysis of left soft palate, hypophonic speech, and swallowing difficulties especially for liquids. No skin vesicular blisters were observed.

Based on such a clinical picture that was not completely clear, the patient underwent laboratory investigations, otorhinolaryngological evaluation, and brain magnetic resonance imaging (MRI).

Brain MRI, repeated twice with and without contrast and even including details on the left facial nerve and the pontocerebellar angle, was thoroughly normal.

A video-laryngoscopy, performed by an otolaryngologist, documented paralysis of the left palate and left vocal cord, pointing to an involvement of glossopharyngeal (IX) and vagus (X) nerves.

The first blood infectious screening showed high levels of IgG against VZV, and increased levels of IgM but not conclusive for acute infection. Moreover, quantification of serum VZV-DNA through PCR proved negative. However, viral serology repeated few days later confirmed the increased IgG antibody level and revealed a further increase of IgM antibodies against VZV, hence documenting a seroconversion. Based on these findings, the patient underwent lumbar puncture, and cerebrospinal fluid analysis through Multiplex-PCR for infectious diseases was positive for VZV DNA.

Accordingly, a diagnosis of multiple cranial neuropathy due to reactivation of VZV (Ramsay Hunt syndrome) was made.

The patient started antiviral therapy with oral Acyclovir (800 mg 5 times a day) and Prednisone (25 mg daily for 10 days) and was discharged home. After 12 days, the infectious disease physicians changed the antiviral therapy with Valacyclovir (1000 mg 3 times a day) for 2 more weeks.

Over a couple of months, the patient gradually improved with a complete recovery of speech and swallowing and an almost full recovery of facial nerve persisting a mild lower face asymmetry. The pain, both temporo-auricular and pharyngeal, had resolved completely after a few days from the initiation of therapy.

## Discussion

The present case may be of interest as it underlines a possible diagnostic challenge that clinicians may hit when facing a common disorder such as peripheral facial palsy. Indeed, clinicians must be aware that Ramsay Hunt syndrome may develop without skin vesicular eruption as well it may be complicated by multiple cranial nerve involvement. It is well known that first infection of VZV causes chickenpox and after VZV remains dormant in sensory ganglia and over time may reactivate spreading within the nerve and causing skin vesicles along the corresponding dermatome. Notably, Ramsay Hunt syndrome is due to reactivation of latent VZV in the geniculate ganglion and accordingly the vesicles develop along the sensory territory of the facial nerve (auditory canal or the auricle). However, Ramsay Hunt syndrome may occur without skin eruption in up to one-third of patients [2]. Our patient presented with a peripheral facial nerve palsy without skin eruption and thus a diagnosis of Ramsay Hunt with respect to an idiopathic facial nerve palsy was not applicable without serology. Moreover, the first serology was not conclusive for confirming a reactivation of VZV as seroconversion had not still occurred.

In most cases, peripheral facial nerve palsy is idiopathic. Such condition is also known as Bell’s palsy and has high prevalence and generally benign prognosis [5].

However, it is crucial to exclude secondary causes of paralysis, as they may have poorer prognosis and require a more aggressive approach. The list of potential causes of facial palsy is wide including infections, tumors, paraneoplastic syndromes, and autoimmune conditions [6–9].

Facial nerve palsy caused by VZV has poorer prognosis than Bell’s palsy, and only half of the patients recover completely [10]. As well the recognition of VZV reactivation is important for properly choosing and starting antiviral therapy. Indeed, it is still debated whether antiviral treatment can be effective in idiopathic facial nerve palsy while antiviral treatment is certainly effective in Ramsay Hunt syndrome, without any apparent difference in efficacy between different antivirals [11]. Instead, steroids, independently from demonstration of viral infection, are highly effective and increase the likelihood of recovery of nerve function in peripheral facial nerve palsy [12, 13].

The second challenge figured out by the present case report was multiple cranial involvement. In fact, our patient in addition to signs of peripheral facial nerve palsy showed signs and symptoms consistent with the involvement of vestibulocochlear (gait instability, nystagmus), glossopharyngeal, and vagus (left soft palate paralysis, dysphagia, vocal cord paresis with hoarseness) nerves.

Involvement of the VIII cranial nerve (vestibulocochlear nerve) is well documented in literature and has been related to anatomical proximity of facial (VII) and vestibulocochlear (VIII) nerves [14].

However, the mechanism of diffusion of VZV to other cranial nerves remains to be elucidated. Some authors postulated VZV dissemination beyond geniculate ganglion, which could explain the involvement of additional cranial nerves via anatomical communications [15]. Other authors instead suggested that inflammation may spread along the vascular system through ascending pharyngeal artery that supplies more cranial nerves (e.g., IX and X) [16].

In conclusion, this case highlights the possible challenges in diagnosing Ramsay Hunt Syndrome, when an atypical presentation occurs. Clinicians should consider the variable clinical spectrum of Ramsay Hunt syndrome when facing an apparently idiopathic peripheral facial nerve palsy. Antiviral therapy is effective in VZV reactivation for recovery of nerve function [17].
